# Analysing functional implications of differences in left ventricular morphology using statistical shape modelling

**DOI:** 10.1038/s41598-022-15888-y

**Published:** 2022-11-10

**Authors:** Froso Sophocleous, Lucy Standen, Gemina Doolub, Reem Laymouna, Chiara Bucciarelli-Ducci, Massimo Caputo, Nathan Manghat, Mark Hamilton, Stephanie Curtis, Giovanni Biglino

**Affiliations:** 1 Bristol Medical School, Faculty of Life Sciences, Bristol Heart Institute, Bristol Royal Infirmary, University of Bristol, Upper Maudlin Street, Bristol, BS2 8HW UK; 2 Bristol Heart Institute, University Hospitals Bristol and Weston NHS Foundation Trust, Bristol, UK; 3 National Heart and Lung Institute, Imperial College London, London, UK; 4 Royal Brompton & Harefield Clinical Group, Guy’s and St Thomas’ NHS Foundation Trust, London, UK

**Keywords:** Computational biology and bioinformatics, Cardiology

## Abstract

Functional implications of left ventricular (LV) morphological characterization in congenital heart disease are not widely explored. This study qualitatively and quantitatively assessed LV shape associations with a) LV function and b) thoracic aortic morphology in patients with aortic coarctation (CoA) with/without bicuspid aortic valve (BAV), and healthy controls. A statistical shape modelling framework was employed to analyse three-dimensional (3D) LV shapes from cardiac magnetic resonance (CMR) data in isolated CoA (n = 25), CoA + BAV (n = 30), isolated BAV (n = 30), and healthy controls (n = 25). Average 3D templates and deformations were computed. Correlations between shape data and CMR-derived morphometric parameters (i.e., sphericity, conicity) or global and apical strain values were assessed to elucidate possible functional implications. The relationship between LV shape features and arch architecture was also explored. The LV template was shorter and more spherical in CoA patients. Sphericity was overall associated with global and apical radial (*p* = 0.001, R^2^ = 0.09; *p* < 0.0001, R^2^ = 0.17) and circumferential strain (*p* = 0.001, R^2^ = 0.10; *p* = 0.04, R^2^ = 0.04), irrespective of the presence of aortic stenosis and/or regurgitation and controlling for age and hypertension status. LV strain was not associated with arch architecture. Differences in LV morphology were observed between CoA and BAV patients. Increasing LV sphericity was associated with reduced strain, independent of aortic arch architecture and functional aortic valve disease.

The ability of the heart to change its shape and function in order to maintain normal cardiac output has been characterized as “remodelling”, which can either be adaptive or maladaptive, acute or chronic^1^. Left ventricular (LV) morphology has been proposed as an important marker of remodelling and related to clinical outcomes, with adverse remodelling being a strong predictor of cardiovascular disease^2–4^. The LV geometry plays a critical role in the mechanics of the heart, and as early as 1892, Woods used a spherical model to quantitatively assess this relationship^5,6^. Three-dimensional (3D) patient-specific anatomical information can be derived by non-invasive imaging techniques, such as cardiovascular magnetic resonance (CMR) imaging. This allows further exploration of LV morphology using tools such as statistical shape modelling (SSM), which our group has previously applied to capture apical ballooning in Takotsubo cardiomyopathy^7^ and other studies used to assess aortic and LV remodelling in scenarios such as myocardial infarction, AAo thoracic aneurysm and aortic stenosis^8–11^. Here, we apply this methodology to congenital heart disease (CHD), particularly to aortic coarctation (CoA) and bicuspid aortic valve (BAV) disease, which have a prevalence of 3/10,000 and 1–2% respectively^12^. Patients with CoA can have BAV in up to 85% of cases, while both diseases can result in aortic vasculopathy, which is associated with morbidity and mortality in young patients with CHD^12,13^.

To our knowledge, only one study has described LV morphology in adults with CoA or BAV, assessing LV sphericity pre- and post-stent implantation in CoA patients^14^. Clinical observations suggest that CoA patients tend to have rounder LVs, but this has not been assessed quantitatively, nor have possible functional implications of this characteristic been explored. Thus, this study aims to assess LV morphology in patients with CoA and/or BAV, using CMR-derived sphericity and conicity indices, and by means of SSM^15^ thus providing novel observations including 3D assessment. Morphological quantification through SSM allows exploring possible previously unreported associations between morphological features and functional implications, such as differences in CMR-derived global and apical strain indices across different LV morphologies. In addition, previous work from our group suggested potentially unfavourable aortic arch configurations in patients with BAV and repaired CoA^11^ and so this study also aimed to explore possible associations between LV morphological features and aortic arch architecture.

## Methods

### Patient population

This was a retrospective single-centre study, screening patients who underwent clinical cardiovascular magnetic resonance (CMR) examination at a large tertiary centre between 2007 and 2020. Data were acquired at 1.5 T (Avanto, Siemens Healthineers, Erlangen, Germany). Patients for the morphological 3D analysis were selected from databases of n = 521 BAV patients, n = 633 CoA patients and n = 435 patients without structural heart disease (serving as healthy controls). Primary exclusion criteria were: connective tissue disorders (including Marfan, Turner and Ehlers Danlos syndromes), any concomitant either complex or moderate CHDs (including Shone’s complex, tetralogy of Fallot, transposition of the great arteries and Ebstein’s anomaly), unrepaired CoA, pseudo-CoA, Kawasaki disease, previous operation of aorta (apart from CoA repair) and/or valve replacement, valvotomy or aortic arch reconstruction, reduced ejection fraction (EF) < 40%, and pregnancy. Further exclusion criteria included sub-optimal image quality or absence of cine CMR images of the left ventricle required for 3D shape reconstruction. We selected patients between 19 and 75 years of age and created four groups: i.e., isolated CoA, CoA + BAV, isolated BAV and healthy controls. Sample of n = 25–30 was arbitrarily deemed as sufficiently large, based on SSM literature, to explore differences between groups. Ethical approval was waived by the University Hospitals Bristol & Weston. Research & Innovation (R & I) Department for this study. Informed consent was given by all patients for research use of images at the time and as part of the CMR scan consent. The study was carried out in accordance to local protocols and regulations.

Demographic variables gathered from clinical records included sex, age and body mass index. Anatomical variables included aortic valve morphology, classified according to the pattern of coronary leaflet non-separation. Functional variables included presence of functional aortic valve disease [i.e., aortic regurgitation (AR) and/or aortic stenosis (AS), classified according to the European Association of Echocardiography^16^], presence and severity of CoA as classified in the literature^17^, history of hypertension, and CMR-derived EF, end-diastolic volume (EDV), end-systolic volume (ESV), stroke volume (SV), and LV mass in end-diastole, collected from clinical CMR reports. The ECG gating—CMR acquisition parameters for the LV analysis were as follows: echo time (TE) = 1.14 ms, effective temporal resolution = 40.05 ms, 25 phase reconstructions giving a reconstructed temporal resolution of 40 ms at a heart rate of 60 bpm, slice thickness = 8 mm, field of view (adapted to patient size) typically 350 × 284 mm, acquisition matrix 192 × 156 mm, giving a typical pixel size 1.8 mm × 1.8 mm. The whole of the LV was examined in the short axis with the basal slice planned on the mitral valve annulus from the long axis images. If a further slice was needed to show the whole of the LV outflow tract this was added. Post gadolinium aortic angiography (gadovist) 0.1–0.2 ml/kg as per radiographic/physician preference slice thickness 1 mm. Acquisition matrix 448 × 294 with field of view adapted to give an ~ 1 × 1 × 1 mm voxel.

### Morphological analysis

**Figure 1:**
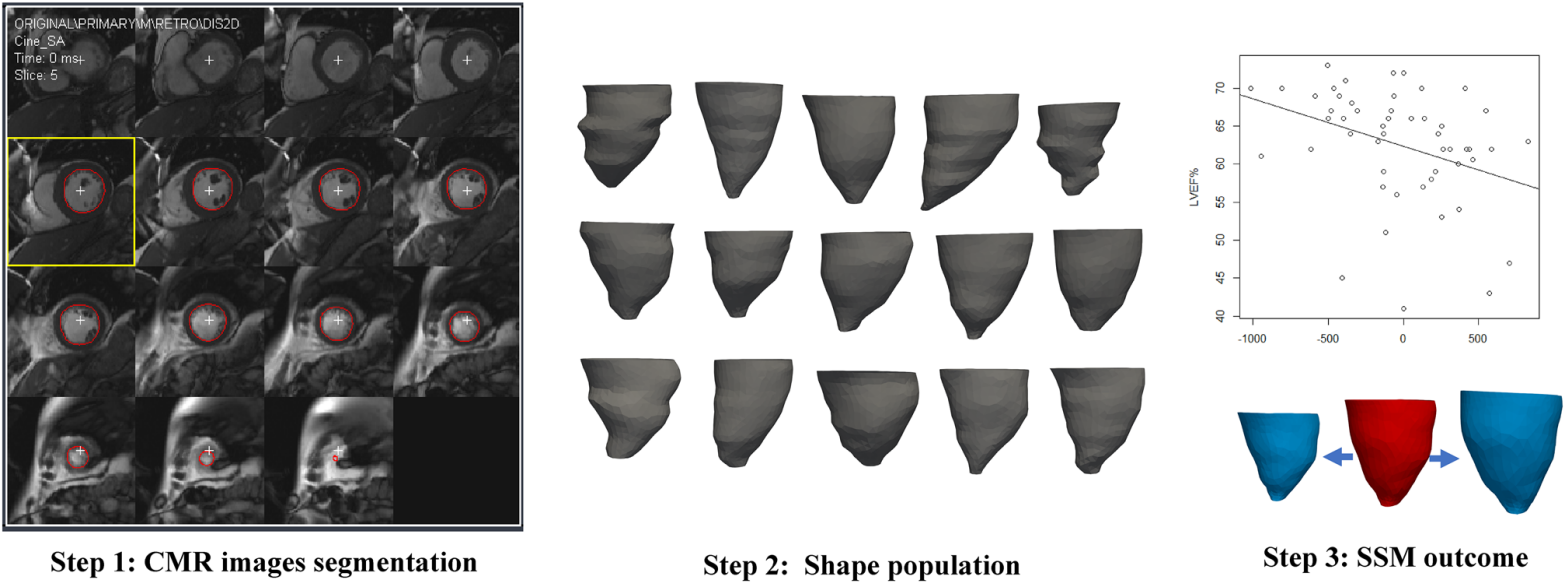
Statistical shape modelling (SSM) framework. Step 1: Cardiovascular magnetic resonance (CMR) cine short axis images segmentation to create 3D volume meshes of LV endocardium (in red) at end-diastole using Segment software; Step 2: Indication of the reconstructed shape population; Step 3: A shape mode showing a deformation as being correlated to LVEF%, exemplifying the feasibility of statistical analysis to capture parameters of interest.

A SSM framework was applied to assess the LV shape, as a primary aim of this study (the methodological workflow is summarised in Fig. 1). Fundamentally, SSM allows studying (a) the three-dimensionality of the shape, taking into account the whole burden of information provided by the CMR scan; (b) the exploration of the average shape in a population along with the different shape deformations; and (c) the statistical association(s) between shape features and demographic/clinical/functional parameters of interest. More technical SSM details are reported elsewhere^18^. Cine images in short-axis view were collected for all cases and segmented creating 3D volume meshes of LV endocardium at end-diastole using open-source software (Segment v3.0, Medviso, Lund, Sweden). The whole short axis stack was used for the LV reconstruction. A consistent number of image-stacks was reconstructed per ventricle to include all the anatomical features from the LV apex to the base. The apex was consistently reconstructed up to the point that it was visible. The 3D shapes were then imported into Mimics (Mimics Research v.21.0, Materialise NV, Leuven, Belgium) in stl format, where they were consistently cut at the apex and base, uniformly remeshed using a factor of 4 mm (3matic Research v.13.0, Materialise), and registered on top of each other based on the barycentre to reduce possible alignment bias and speed-up the analysis. The 3D meshes were then imported into Deformetrica software (http://www.deformetrica.org) to compute the average shape of the population, or ‘template’, as well as the shape variation around it, or ‘shape modes’^18^. Key analysis parameters were a) model stiffness, set at 35 mm (λ_diffeos_ parameter, bigger values result in “stiffer”, i.e., less elastic, deformations that capture more global shape features) and b) model resolution, set at 11 mm (λ_surface_ parameter, bigger values result in neglecting small shape features). The shape templates provide qualitative data, while the shape vectors provide quantitative data for dominant morphological features (or shape modes). Shape modes were extracted from principal component analysis in order to statistically correlate shape deformations with parameters of interest, i.e., sphericity and conicity indices, global and apical strains, and aortic arch shape. On top of the global analysis, all groups were separately processed, using the same parameters, allowing for visual differences to be displayed.

**Figure 2:**
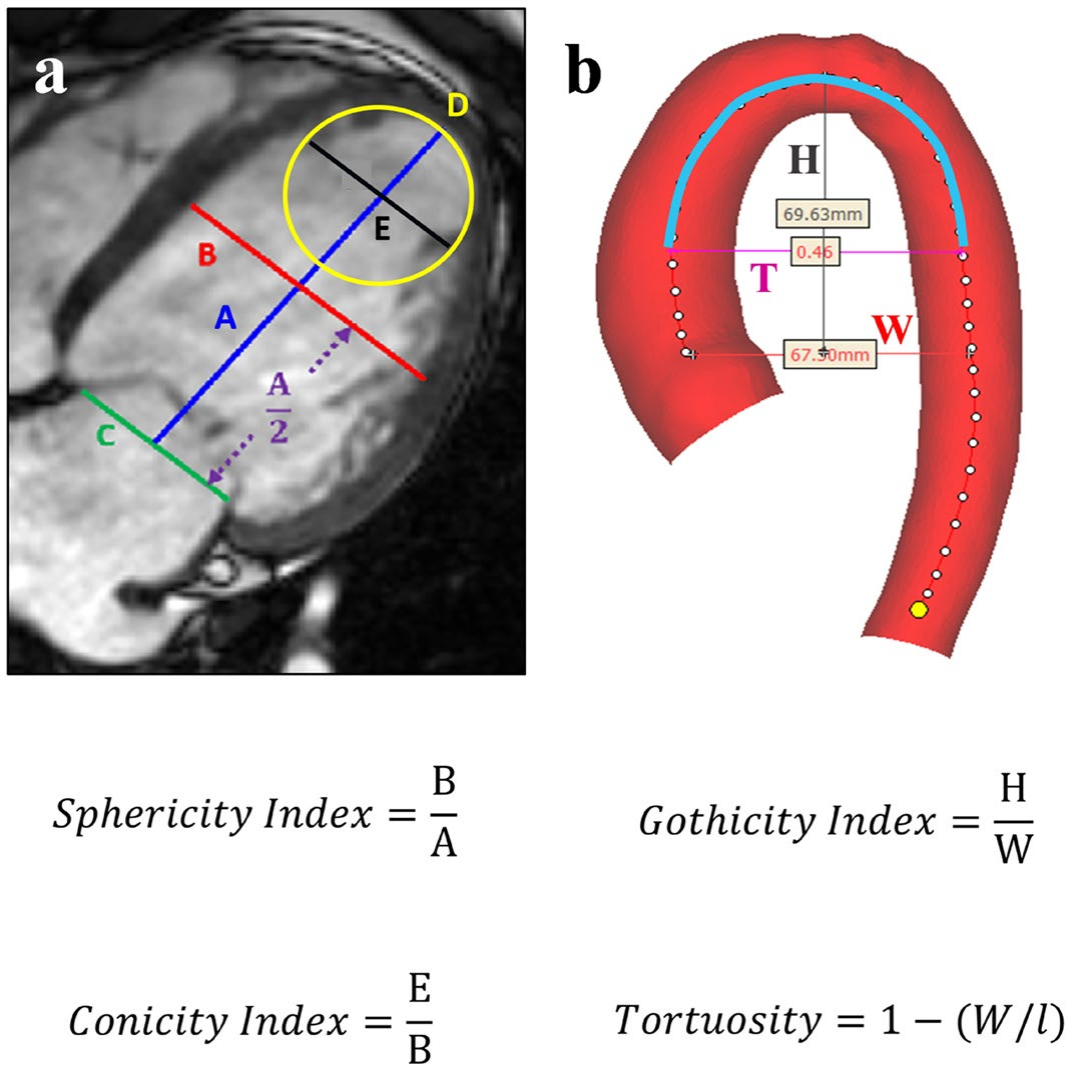
Morphometric measurements. (**a**) Calculation of LV sphericity as short (B) to long (A) axis ratio, with short axis set as the perpendicular intersect of the long axis mid-point (A/2) while long axis being the distance from the mitral valve mid-point (C) to apex, and calculation of LV conicity as apical (E) to short axis ratio, with apical axis being the diameter of the best fitting sphere (D) (calculations were taken directly on the scans). (**b**) Calculation of aortic gothicity as height to width ratio (H/W) [The aortic arch width was measured as the perpendicular distance from the centreline at the sinotubular junction level to the centreline at the mid-descending aorta. The height of the arch was measured as the maximum vertical distance from the width measurement to the highest point of the centreline in the arch], and aortic tortuosity as 1-(W/l) with l being the length of the aortic arch as shown by T (highlighted in blight-blue on the centreline). T shows the endpoints set to indicate the aortic arch defined by using the pulmonary artery as a landmark (calculation were taken using Mimics software).

Sphericity and conicity measurements were averaged from CMR cine 2- and 4-chamber imaging views. Sphericity was measured as short to long axis ratio, while conicity was measured as apical to short axis ratio, with apical axis being the diameter of the best fitting sphere to the apex as described in^15^, (see Fig. 2a).

In order to assess possible associations between arch architecture and LV morphology, 3D whole heart or angiographic sequences were used to reconstruct 3D aortic arch models (Mimics). This was carried out in a subset of patients (67/110: isolated CoA n = 23, CoA + BAV n = 30, isolated BAV n = 14, and healthy controls N/A) that had suitable 3D CMR sequences; this excluded all the healthy controls. The 3D aortic models were consistently cut perpendicularly at the subannular level and at the level of diaphragm. The brachiocephalic, left common carotid, left subclavian and coronary arteries were excluded because the study focused on examining the aorta alone. Earlier work showed that errors from the manual segmentation process are < 1 mm i.e., less than the voxel size^19^. The 3D aortic models were used to measure aortic gothicity (based on height/width ratio, H/W) and aortic tortuosity (automatically calculated using Vascular Modelling Toolkit, Orobix, Bergamo, Italy) (see Fig. 2b), as previously described^11,20^. The rationale for assessing potential associations between arch architecture and the underlying LV morphology is that, mechanically, a more complex (e.g., more Gothic and more tortuous) arch geometry could represent a less hemodynamically favourable scenario and potentially reflect on the LV. The LV shapes were reconstructed at end-diastole and the aortas were reconstructed in peak-systole, to capture the respective largest deformations.

### Deformation analysis

Regional tissue tracking features in three directions (longitudinal, radial, and circumferential) were automatically computed on a 16-segment model and averaged to provide global peak longitudinal strain (GLS), global peak radial strain (GRS), global peak circumferential strain (GCS). The endocardial and epicardial contouring were carefully delineated from the cine images throughout the cardiac cycle. The feature tracking analysis was performed in CVI42 (Circle Cardiovascular Imaging, Calgary, AB, Canada). Intra/inter-observer variability of the CMR-derived strain measurements has already been established^21^. Global longitudinal strain was calculated from 2-, 3- and 4-chamber views and global radial and circumferential strains were calculated from three short axis slices (basal, mid and apical). The apical/basal segments were selected as the most apical/basal segment in the short axis with well-defined circular contour (endocardium and epicardium). The mid-myocardium was selected as the slice mid-way between basal and apical with two clear papillary muscles Endocardial and epicardial contours were derived to calculate all strain measurements.

### Statistical analysis

Statistical analysis was run with R Studio (Vienna, Austria). Group differences for continuous variables were tested using ANOVA or Kruskal–Wallis test (with Dunn’s test post hoc) depending on the normality of the distribution. The reproducibility of LV 3D reconstructions was assessed on 20% of the study population, randomly selecting cases for re-segmentation after 12 months and assessing median % difference in mesh surface area, intraclass correlation coefficient (ICC) for reproducibility and Bland Altman analysis for bias. Linear regression analysis was performed to verify the association between variables, including between SSM-derived shape modes and demographic, anatomical and functional variables. Multiple regression analysis was run to assess the effect of aortic arch architecture (i.e., tortuosity and gothicity), AR, AS, hypertension, age, BMI, and sex on the relationship between LV morphology and strain. For the purpose of the analysis and considering the available sample size, AR and AS severity were treated as categorical variables (none or mild = 0, moderate and severe = 1). Alpha was set at 0.05.

### Ethics approval and consent to participate

In light of the nature of the study, ethical approval was not required by the local Research & Innovation Department.

## Results

Patient demographic and clinical characteristics are reported below in Table 1 and CMR measurements are reported in Table 2. The isolated CoA group had higher prevalence of history of hypertension (*p* = 0.03). The majority of BAV patients had right-left leaflet non-separation. Also, CoA + BAV patients had increased CoA severity compared to isolated CoA (moderate or severe CoA in 37% vs 21%, *p* = 0.01) and, overall, very few patients presented with severe AR or AS. All groups had normal EF around 65%, and patients with BAV had larger LV volumes and higher LV mass (Table 2). Interestingly, patients with CoA showed higher sphericity index (*p* = 0.02) whilst no group difference was found for arch gothicity or tortuosity (*p* = 0.28 and *p* = 1, respectively).

**Table 1:** Patient demographic and clinical characteristics.

	Isolated CoA (N = 25)	CoA + BAV (N = 30)	Isolated BAV (N = 30)	Controls (N = 25)	*p* values
Sex (n, % M)	12, 48%	16, 53%	16, 53%	12, 48%	*p* = 0.96
Age (years)	37 ± 12, (19–66)	43 ± 8, (31–57)	46 ± 14, (20–75)	45 ± 11, (27–64)	*p* = 0.03
BMI (kg/m^2^)	27 ± 6, (20–42)	27 ± 5, (17–38)	26 ± 5, (17–39)	26 ± 4, (21–34)	*p* = 0.97
Presence of hypertension (n, %)	18, 72%	11, 37%	13, 48%	–	*p* = 0.03
Valve Phenotype (n, %)	–	RL: 26, 87% RNC: 3, 10% LNC: 1, 3%	RL: 19, 63% RNC: 11, 37% LNC: 0, 0%	–	
CoA severity, (n, %)	None^**§**^: 11, 44% Mild: 9, 36% Moderate: 1, 4% Severe: 4, 16%	None^**§**^: 12, 40% Mild: 7, 23% Moderate: 3, 10% Severe: 8, 27%	–	–	
AR severity, (n, %)	None: 22, 88% Mild: 3, 12% Moderate: 0, 0% Severe: 0, 0%	None: 14, 47% Mild: 13, 43% Moderate: 2, 7% Severe: 1, 3%	None: 7, 24% Mild: 15, 50% Moderate: 5, 17% Severe: 3, 10%	None: 25, 100% Mild: 0, 0% Moderate: 0, 0% Severe: 0, 0%	
AS severity, (n, %)	None: 25, 100% Mild: 0, 0% Moderate: 0, 0% Severe: 0, 0%	None: 21, 70% Mild: 6, 21% Moderate: 2, 7% Severe: 1, 3%	None: 15, 50% Mild: 3, 10% Moderate: 8, 28% Severe: 4, 14%	None: 25, 100% Mild: 0, 0% Moderate: 0, 0% Severe: 0, 0%	

*AR* aortic regurgitation, *AS* aortic stenosis, *RL* right-left leaflet non-separation, *RNC* right-non coronary leaflet non-separation, *LNC* left-non coronary leaflet non-separation, *BMI* body mass index. None^**§**^ = patients with repaired CoA and no residual narrowing.

**Table 2:** CMR measurements.

	Isolated CoA (n = 25)	CoA + BAV (n = 30)	Isolated BAV (n = 30)	Controls (n = 25)	*p* values
**Ventricular measurements**					
EF%	65 ± 8	64 ± 5	65 ± 8	64 ± 4	*p* = 0.91
EDV (ml/m2)	76 ± 19	107 ± 48	119 ± 48	75 ± 10	*p* < 0.0001
ESV (ml/m2)	27 ± 8	39 ± 15	42 ± 19	27 ± 5	*p* < 0.0001
SV (ml)	51 ± 16	68 ± 35	77 ± 32	49 ± 7	*p* < 0.001
LV mass (g/m2)	62 ± 26	84 ± 47	106 ± 54	55 ± 11	*p* < 0.001
Global Longitudinal Stain (%)	− 17.1 ± 2.1	− 17.5 ± 2.0	− 16.5 ± 2.3	− 18.2 ± 2.2	*p* = 0.02
Global Radial Strain (%)	35.5 ± 8.2	35.7 ± 6.0	33.6 ± 8.0	37.0 ± 6.2	*p* = 0.16
Global Circumferential Strain (%)	− 19.9 ± 2.7	− 20.1 ± 2.2	− 19.5 ± 2.4	− 20.4 ± 1.9	*p* = 0.49
Apical Radial Strain (%)	47.4 ± 14.4	38.1 ± 10.5	43.9 ± 12.2	47.4 ± 12.8	*p* = 0.03
Apical Circumferential Strain (%)	− 23.2 ± 3.7	− 18.3 ± 10.7	− 21.3 ± 8.2	− 23.8 ± 3.0	*p* = 0.02
LV Sphericity Index	0.62 ± 0.08	0.62 ± 0.08	0.59 ± 0.06	0.57 ± 0.06	*p* = 0.02
LV Conicity Index	0.52 ± 0.07	0.51 ± 0.07	0.51 ± 0.06	0.47 ± 0.05	*p* = 0.05

	Isolated CoA (n = 23)	CoA + BAV (n = 30)	Isolated BAV (n = 14)	Controls (n = 0)	*p* values
**Aortic measurements**					
Arch Gothicity	1.19 ± 0.17	1.28 ± 0.23	1.32 ± 0.30	–	*p* = 0.28
Arch Tortuosity	0.46 ± 0.07	0.46 ± 0.07	0.46 ± 0.06	–	*p* = 1

Controls lacked the sequence for 3D aortic reconstructions and therefore arch measurements were not available.

*EF* ejection fraction, *EDV* end-diastolic volume, *ESV* end-systolic volume, *SV* stroke volume.

**Figure 3:**
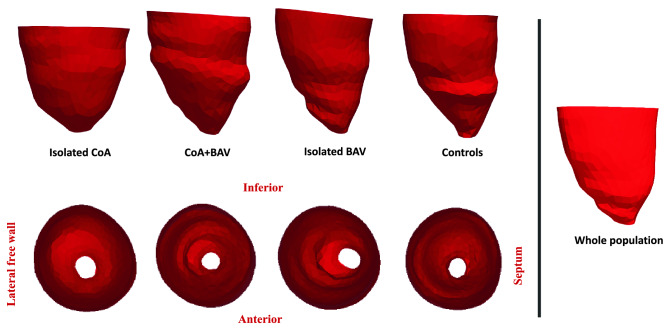
LV shape templates, showing the average 3D configuration of the ventricles. As labelled, the figure illustrates the average shapes of each group separately, both side and top views. The whole population template is also shown.

Three-dimensional LV models were highly reproducible, with a median difference in reconstructions performed 12 months apart of 4% (range 0–9%), excellent reproducibility (ICC = 0.98) and no bias (please refer to Supplementary Material for Bland Altman plot and additional illustrations). Three-dimensional templates, calculated for the whole population and the four groups separately, are shown in Fig. 3, with atlas error ≤ 0.4 mm. As it can be qualitatively appreciated, the template for isolated CoA patients is the shortest and more spherical compared to the others, followed by BAV-CoA and then isolated BAV, compared to the more elongated healthy controls.

**Figure 4:**
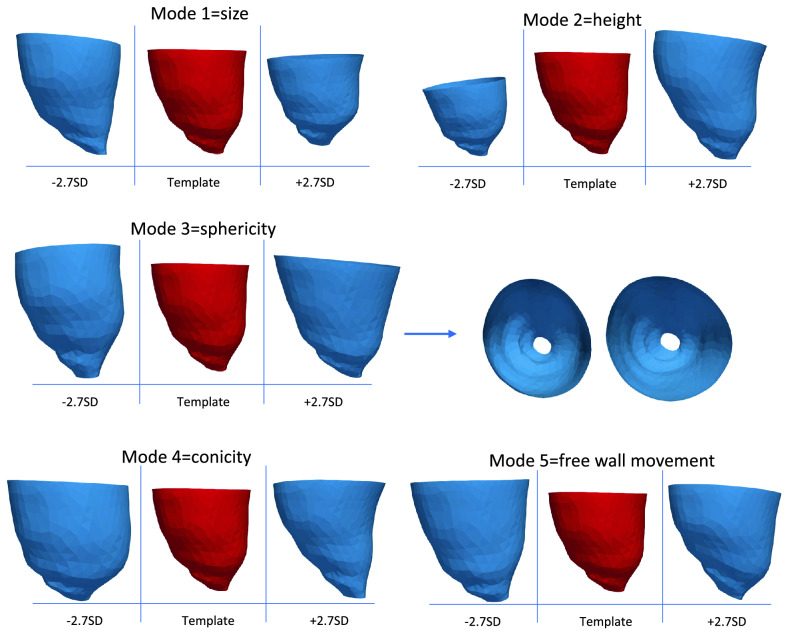
Shape modes. Illustration of the five computed shape modes capturing LV variation (± 2.7 SD from the mean configuration, representing the shape mode extremities, or more extreme anatomic features in this population). For each mode, it is indicated what the mode overall captures, e.g., Mode 1 = size. SD = standard deviation.

**Figure 5:**
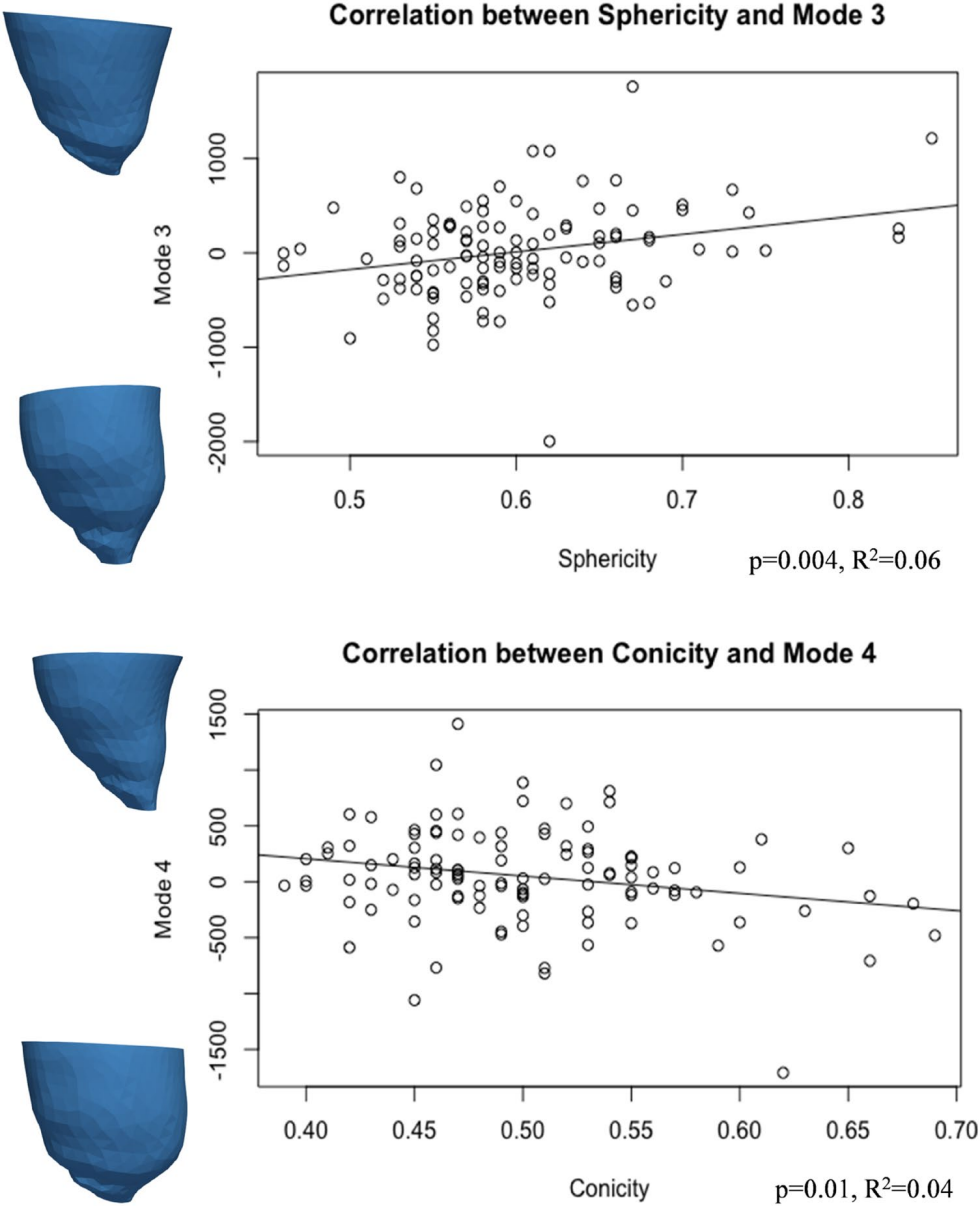
Correlations for shape modes 3 and 4 and corresponding morphometric LV measurements (sphericity and conicity). Sphericity is significantly positively correlated with Mode 3. SD = standard deviation.

The first five principal component analysis shape modes (Fig. 4) represented 54% of the overall shape variability in the population (Table 3), and hence the corresponding shape vectors were used for statistical analyses. Different modes captured different morphological features, after careful visual assessment and correlation with morphometric measurements (Fig. 5). Dominant shape features included: overall LV size (Mode 1), height (Mode 2), sphericity (Mode 3), conicity (Mode 4), and free wall movement (Mode 5). Group differences for shape modes confirmed that the isolated CoA group had the shortest and more spherical LV shape (Mode 1, *p* = 0.02; Mode 2, *p* = 0.01; Mode 3, *p* < 0.001).

**Table 3:** Shape modes (M) with individual and cumulative contribution in LV variation (“Inertia %”).

Mode	Inertia%	Cumulative inertia%
M1	14.37	14.37
M2	13.64	28.01
M3	9.83	37.84
M4	8.37	46.21
M5	7.33	53.54

This represents the % shape variability captured by each shape mode individually and cumulatively.

**Figure 6:**
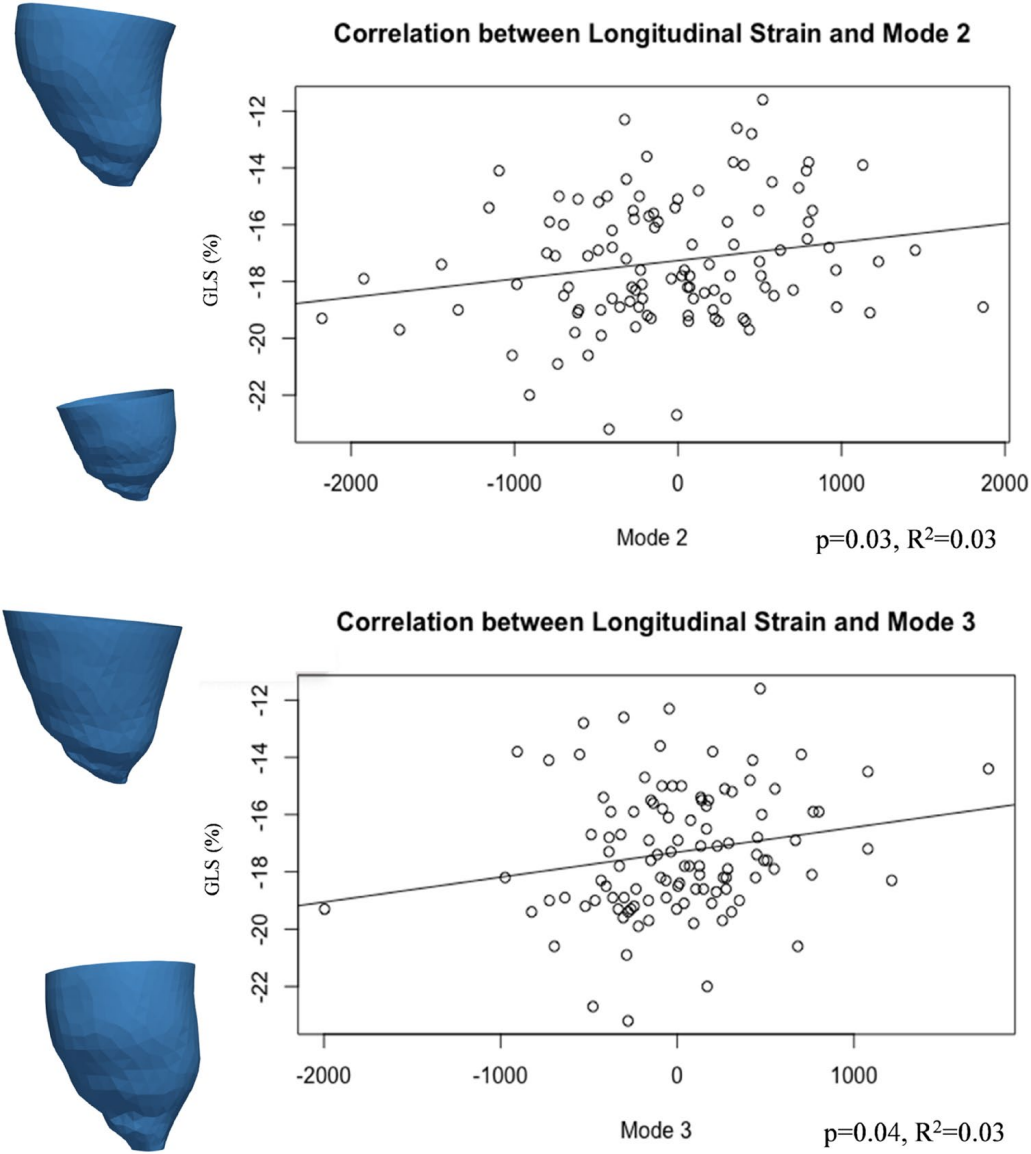
GLS was associated with Modes 2 and 3, showing that increased height and sphericity correlate with worse LV function.

**Figure 7:**
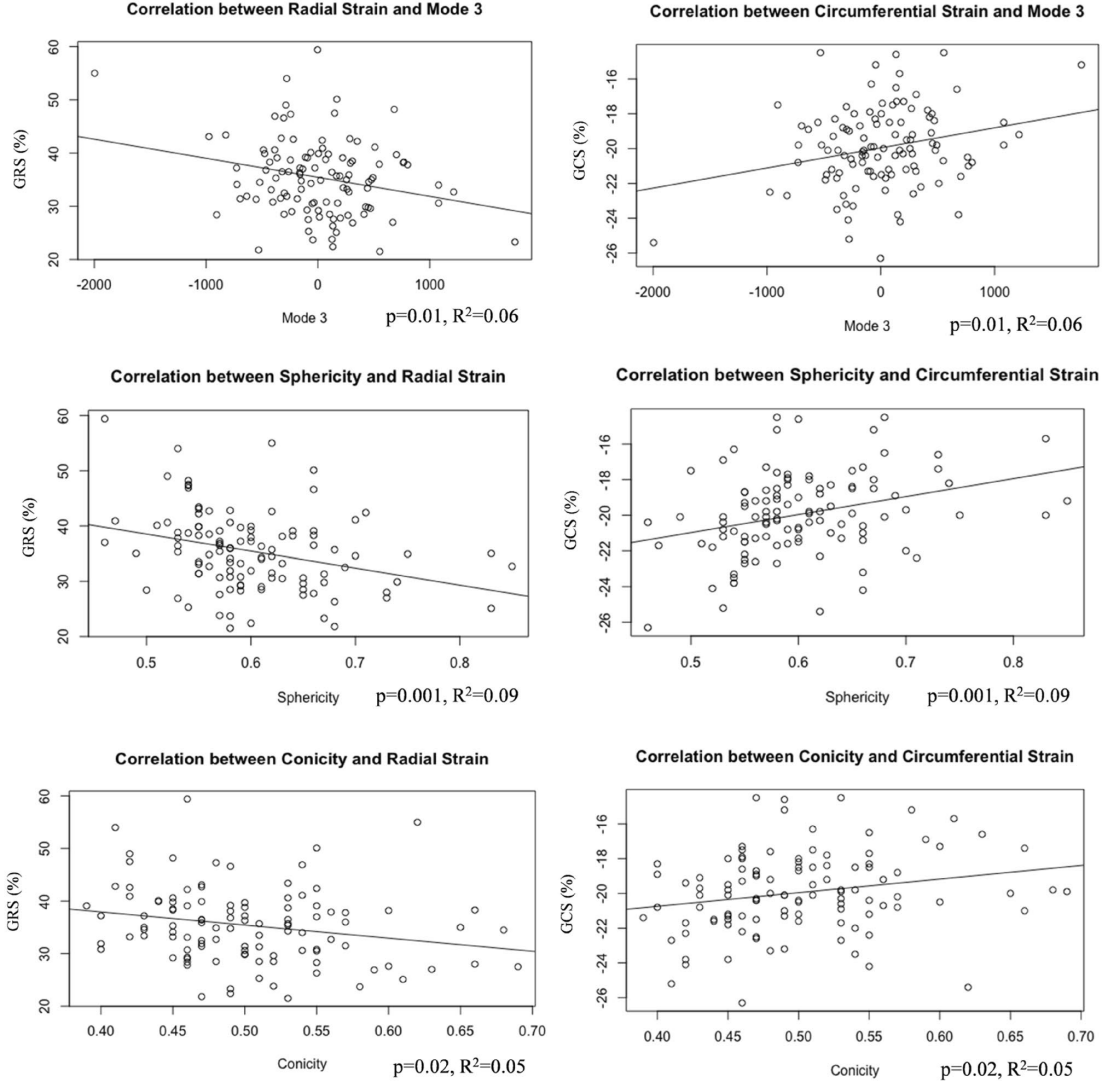
LV shape correlation with function. This figure illustrates the association between GRS and GCS with Mode 3, sphericity and conicity.

Modes 1 and 2 correlated with ventricular volumes (Mode 1 and ESV: *p* = 0.05, R^2^ = 0.04; Mode 2 and ESV: *p *< 0.001, R^2^ = 0.14; Mode 2 and EDV: *p* < 0.001, R^2^ = 0.15). Interestingly, GLS correlated with both Mode 2 and Mode 3 (Fig. 6), both separately and in a multiple regression(*p* = 0.01), suggesting that there are functional implications of rounder and shorter LVs (as observed in CoA patients). Furthermore, Mode 3 was negatively correlated with all global strain measurements [i.e. GLS (*p* = 0.04, R^2^ = 0.04), GRS (*p* = 0.01, R^2^ = 0.06) and GCS (*p* = 0.01, R^2^ = 0.06)], and sphericity correlated with both global and apical radial (*p* = 0.001, R^2^ = 0.09; *p* < 0.0001, R^2^ = 0.17) and circumferential (*p* = 0.001, R^2^ = 0.10; *p* = 0.04, R^2^ = 0.04) strain, overall showing that the more spherical LVs are associated with reduced strain indices. Also, conicity was correlated with GRS (*p* = 0.02, R^2^ = 0.05) and GCS (*p* = 0.02, R^2^ = 0.05) (Fig. 7).

When assessing the association between sphericity and strain indices in a multiple regression model accounting for the presence of AR and AS, only sphericity was significantly associated with GRS (*p* = 0.01), GCS (*p* = 0.01) and apical radial strain (*p* < 0.0001). When assessing the association between sphericity and strain indices in a multiple regression model accounting for aortic arch gothicity and tortuosity, only sphericity was associated with strain (*p* = 0.05 for GRS; we also noted *p* = 0.06 for GCS). On the same line, controlling for age and hypertension status, the association between M3 (sphericity) and global strain indices remained significant (GRS: *p* = 0.03, R^2^ = 0.06, and GCS: *p* = 0.02, R^2^ = 0.1). The same was observed for CMR sphericity measurements and global strain indices (GRS: *p* = 0.03, R^2^ = 0.06, and GCS: *p* = 0.02, R^2^ = 0.1). Also, controlling for age and hypertension, a significant association was confirmed between sphericity and apical strain indices (*p* < 0.001, R^2^ = 0.17 for apical radial strain and *p* = 0.01, R^2^ = 0.08 for apical circumferential strain). Similarly, when assessing the association between sphericity and strain indices in a multiple regression model accounting for BMI and sex, only sphericity was significantly associated with GRS (*p* = 0.002), GCS (*p* = 0.003) and apical radial strain (*p* < 0.0001). Finally, when considering arch architecture, tortuosity and gothicity measurements as derived from 3D aortic shapes showed no group differences (Table 2). Whilst not all patients had 3D aortic models and hence tortuosity data, a weak positive correlation was found between tortuosity and sphericity (*p* = 0.02, R^2^ = 0.08).

## Discussion

Left ventricular remodelling is either an adaptive response to ageing or occurs due to exposure to cardiovascular disease risk factors and myocardial injury^22^. LV sphericity index has been used in the literature, in Takotsubo cardiomyopathy and chronic aortic regurgitation cohorts, as a reproducible bedside echocardiographic measure of geometric changes and a predictor of poor LV function^23,24^. As LV sphericity index fails to detect regional apical abnormalities, a conicity index had also been introduced^15^. Statistical shape modelling is a novel tool to qualitatively and quantitatively assess geometric and functional patterns of the heart that cannot be fully captured by traditional morphometric imaging measurements. A challenge to test shape characterization in patients with myocardial infarction from the Cardiac Atlas Project has been previously carried out to provide additional information over standard clinical measures, but without in-depth interpretation of shape variations^1^. Also, LV shape in a small sample of CoA patients pre- and post-stent was compared to normal patients from the Cardiac Atlas Project to further describe LV remodelling through cardiac shape quantification^14^.

This study focused on CMR LV shape characterization for the first time in four different groups, i.e., isolated CoA, CoA + BAV, isolated BAV and healthy controls. A computational analysis was performed to compute LV average and shape deformations within the population using a methodological framework previously described by our team and colleagues^7,18^. Qualitative assessment of the group templates showed that patients with isolated CoA had a shorter and more spherical configuration on average, also quantitatively confirmed by a higher sphericity index. Importantly, it was found that such a configuration appeared to correlate with functional implications, as revealed by significant associations between increasing sphericity and reduced strain indices (both global and apical).

The sphericity index has been previously found to be associated with LV diastolic dimensions and able to predict systolic dysfunction better than echocardiographic measurements alone in an experimental AR model^25^. In a follow-up study of chronic AR patients to assess LV remodelling and progression, LV volumes and sphericity index were increased whereas GLS, GRS, apical rotation and twist were decreased, independent of drug therapy^24^. Of note, in our study, we observed that the relationship between sphericity and reduced strain continued to remain significant when accounting for the presence of moderate or severe AR and/or AS in a multivariable regression model. Also, in a follow-up study of clinical correlations and prognostic significance of change in LV geometric patterns, higher blood pressure, greater BMI, advancing age and male sex have been found to be key factors for developing abnormal LV geometry, whereas in our study LV geometry was independent of the above factors^26^.

Another factor that could potentially be associated with the morphology and remodelling of the LV is the concurrent variant architecture of the aortic arch. Intuitively a more gothic and tortuous arch would represent higher impedance for the underlying LV, and previous observations in patients with repaired CoA suggested an association between aortic arch gothicity and tortuosity and parameters of LV function (i.e. ejection fraction, volumes and mass)^11,27^. However, in this population comprising patients with CoA and/or BAV, tortuosity and gothicity measurements as derived from 3D aortic shapes showed no group differences. Furthermore, multiple regression revealed that sphericity measures remained significantly associated with LV strain whilst tortuosity and gothicity did not. It should be noted that in this study all patients had a normal EF (~ 65%), whilst in previous studies exploring the functional implications of arch morphology, patients with lower EFs were also included. The functional implications of aortic arch shape remain an interesting point to explore in patients with CHD (including conotruncal anomalies), and previous work has suggested that, from a functional standpoint, it is not associated with a hypertensive response to exercise in patients with repaired CHD^28^. In relation to LV shape changes, it is also interesting to consider that the association between sphericity and radial/circumferential strain components remained significant also when accounting for age and hypertension status. This may be reflecting of our study population. Previous work observed that LV volumes and mass rise in adolescence and decline with age^29^ and LV shape is the same among the age groups, except for young subjects (< 20 years) in which the highest sphericity and lowest conicity were observed^30^.

Strain difference correlated to shape changes might be partially explained by myocardial fiber re-orientation and shortening, although this study lacks this information and future research with diffusion tensor imaging could be very interesting in further investigating the architecture of the LV in these patients. There is a growing body of evidence showing that LV strain is not only a more sophisticated and thus more useful measure of LV systolic function, but that it is also more reproducible and relates to prognosis in a variety of clinical scenarios^31^. Our analysis revealed a functional implication for the observed morphological differences and therefore it may be possible to use these techniques to identify patients at higher risk of adverse remodelling, which could allow for earlier medical intervention and improved outcomes. Thus, by using this methodological framework on bigger cohort and applying clustering (i.e. patient grouping/categorization) we could then unpack the relationship between morphological and functional element and risk-stratify the groups. Also, future studies can take advantage of such a powerful tool as the atlas-based disease assessment to reveal haemodynamic insights^32^. Changes in myocardial thickness were beyond the scope of this study but could be included in future investigations.

*Limitations* This study has the disadvantages of being a retrospective design with a relatively small number of patients per subgroup. Nevertheless, the overall population (n = 110) is one of the largest described using SSM, with previous studies modelling smaller populations^7,27,33^. Blood pressure data (cuff pressure at the time of CMR) were not available, thus a history of hypertension, as found in clinical notes, was reported instead. Finally, gothicity and tortuosity measurements were not available in a substantial portion of cases, limiting the assessment of the relationship between LV morphology and arch architecture. The observed positive correlation between arch tortuosity and ventricle sphericity is intriguing but it should be noted that this was observed in a subset of cases that mostly had CoA (53/67). These observations could be expanded in the future but a 3D sequence is a necessary requirement for this analysis and this is not necessarily part of the routine clinical imaging protocol.

## Conclusions

This study illustrates computationally-derived LV templates, characterizing LV shape in both CoA and BAV patients for the first time. The analysis revealed an association between increasing LV sphericity and reduced LV strain indices, suggesting a functional implication for the observed morphological differences as well as a possible role for computational techniques to identify patients at higher risk of adverse remodelling.

## Supplementary Information

Supplementary Information 1.

Supplementary Information 2.

## Data Availability

Data will be made available upon request.

## Abbreviations

AR: Aortic regurgitation
AS: Aortic stenosis
BAV: Bicuspid aortic valve
CMR: Cardiovascular magnetic resonance
CoA: Coarctation
CHD: Congenital heart disease
EDV: End-diastolic volume
EF: Ejection fraction
ESV: End-systolic volume
GCS: Global peak circumferential strain
GLS: Global peak longitudinal strain
GRS: Global peak radial strain
LV: Left ventricle
LNC: Left-non coronary
RL: Right-left
RNC: Right-non coronary
SSM: Statistical shape modelling
SV: Stroke volume
