# Supplementary figures and images for: Analysing functional implications of differences in left ventricular morphology using statistical shape modelling
Source: Sci Rep. 2022 Nov 10;12:19163. doi: 10.1038/s41598-022-15888-y (PMC9649786; doi:10.1038/s41598-022-15888-y)

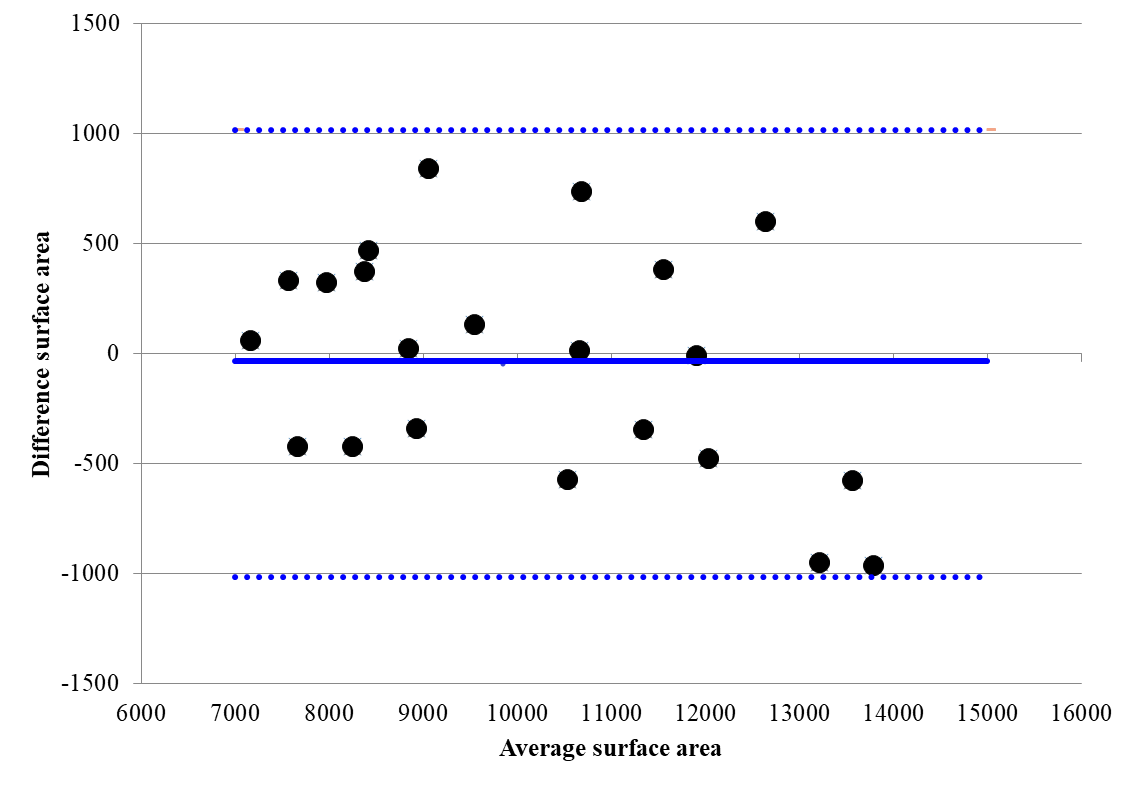

Supplement: Supplementary file 1 — Supplementary Information 1. [file 41598_2022_15888_MOESM1_ESM.tiff]

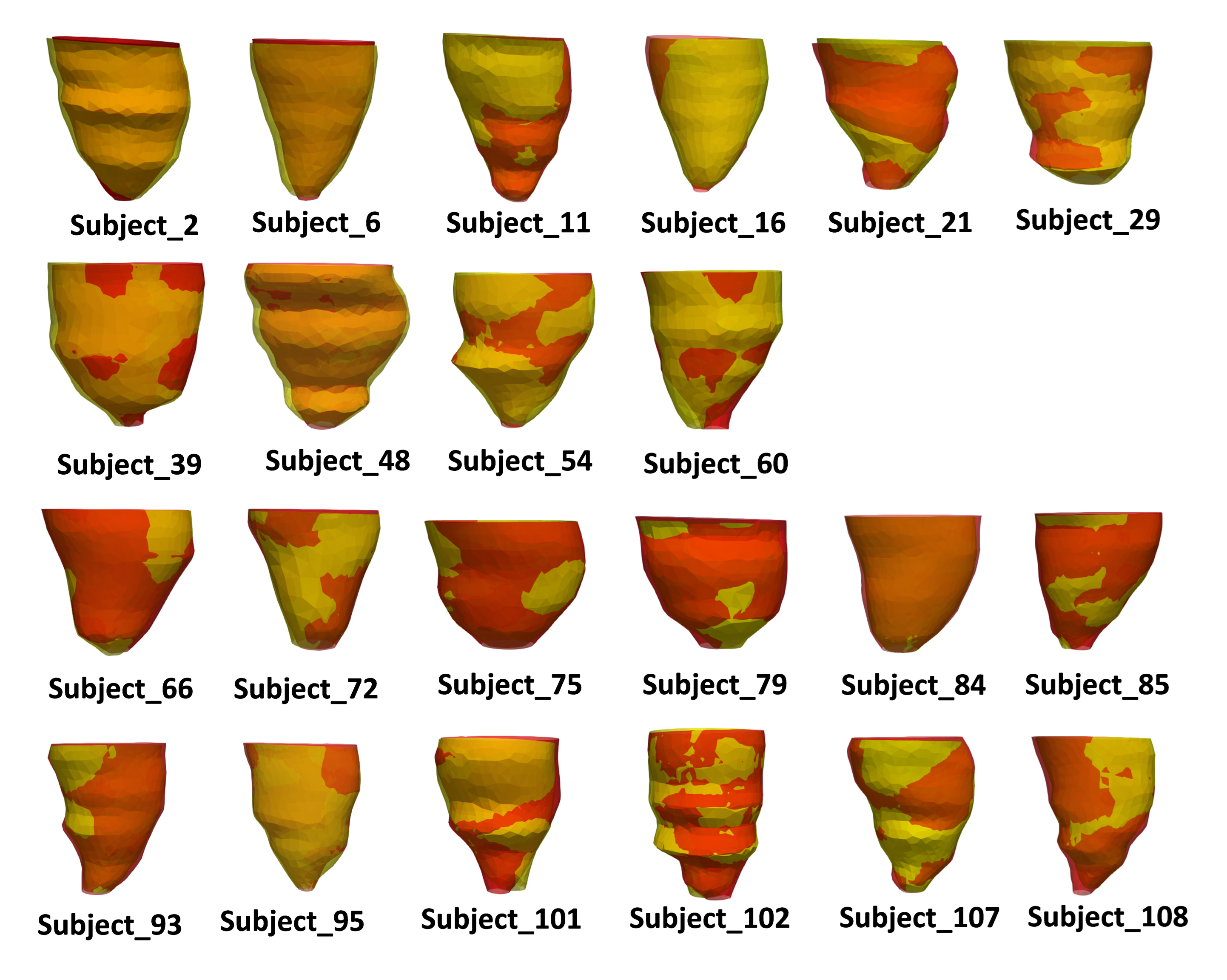

Supplement: Supplementary file 2 — Supplementary Information 2. [file 41598_2022_15888_MOESM2_ESM.tif]
